# Predicting prostate adenocarcinoma patients’ survival and immune signature: a novel risk model based on telomere-related genes

**DOI:** 10.1007/s12672-024-00986-2

**Published:** 2024-06-02

**Authors:** Jiefang Zheng, Jiahui Chen, Hongxiao Li, Yuanchao Li, Weimin Dong, Xianhan Jiang

**Affiliations:** 1 Key Laboratory of Biological Targeting Diagnosis, Therapy and Rehabilitation of Guangdong Higher Education Institutes, Department of Urology, The Fifth Affiliated Hospital of Guangzhou Medical University, Guangzhou, Guangdong China; 2 Clinical College of Acupuncture, Moxibustion, and Rehabilitation, Guangzhou University of Chinese Medicine, Guangzhou, Guangdong China

**Keywords:** Telomere, Risk model, Immune microenvironment, Prostate adenocarcinoma, Immune checkpoint inhibitors

## Abstract

Alterations in telomeres constitute some of the earliest occurrences in the tumourigenesis of prostate adenocarcinoma (PRAD) and persist throughout the progression of the tumour. While the activity of telomerase and the length of telomeres have been demonstrated to correlate with the prognosis of PRAD, the prognostic potential of telomere-related genes (TRGs) in this disease remains unexplored. Utilising mRNA expression data from the Cancer Genome Atlas (TCGA), we devised a risk model and a nomogram to predict the survival outcomes of patients with PRAD. Subsequently, our investigations extended to the relationship between the risk model and immune cell infiltration, sensitivity to chemotherapeutic drugs, and specific signalling pathways. The risk model we developed is predicated on seven key TRGs, and immunohistochemistry results revealed significant differential expression of three TRGs in tumours and paracancerous tissues. Based on the risk scores, PRAD patients were stratified into high-risk and low-risk cohorts. The Receiver operating characteristics (ROC) and Kaplan–Meier survival analyses corroborated the exceptional predictive performance of our novel risk model. Multivariate Cox regression analysis indicated that the risk score was an independent risk factor associated with Overall Survival (OS) and was significantly associated with T and N stages of PRAD patients. Notably, the high-risk group exhibited a greater response to chemotherapy and immunosuppression compared to the low-risk group, offering potential guidance for treatment strategies for high-risk patients. In conclusion, our new risk model, based on TRGs, serves as a reliable prognostic indicator for PRAD. The model holds significant value in guiding the selection of immunotherapy and chemotherapy in the clinical management of PRAD patients.

**Supplementary Information:**

The online version contains supplementary material available at 10.1007/s12672-024-00986-2.

## Introduction

Prostate adenocarcinoma (PRAD) is a prevalent male malignancy, displaying diverse clinical presentations, treatment responses, and recurrence patterns due to its molecular heterogeneity [1, 2]. Precise treatment plans are challenging to formulate for individual patients. Therefore, there is a crucial need to establish reliable prognostic models [3]. Existing models, such as the Gleason scoring system, International Society of Urological Pathology (ISUP) grading system, and American Joint Committee on Cancer (AJCC) TNM staging system, integrate patient characteristics but have limitations [4–6]. Advancements in sequencing technology offer an opportunity to identify critical genes associated with PRAD prognosis, enabling more accurate risk assessment and novel molecular subtyping methods surpassing current staging approaches [7].

Telomeres, which are unique structures located at the ends of chromosomes, consist of highly repetitive DNA sequences and play a crucial role in maintaining the integrity of the genome. In normal cells, telomeres gradually shorten with each cell division, leading to cell cycle arrest or apoptosis [8]. However, tumor cells employ unique mechanisms like telomerase activation and telomerase-independent alternative lengthening telomeres for maintenance [9, 10]. Studies have demonstrated that telomere length changes and maintenance have been linked to tumor survival, proliferation, metastasis, and poor clinical outcomes in various cancers, including PRAD [11–13]. PRAD is characterized by significant telomere shortening associated with genomic instability, chromosomal abnormalities, and oncogene activation. Heaphy et al. found that men with more pronounced intercellular telomere length changes in PRAD tissue or shorter telomeres in cancer-associated stromal cells had a higher risk of disease progression or death [14, 15]. Recent studies also have examined the effects of core components of telomerase, including the telomerase RNA gene TERC and the telomerase reverse transcriptase, on PRAD. Baena et al. observed that increased TERC expression was associated with a poorer prognosis in patients with PRAD [16]. Furthermore, Poos et al. found that the expression levels of PITX1, a transcription factor that regulates TERC, increased with increasing levels of Ki67 in PRAD, indicating a poorer clinical outcome [17, 18].

However, alterations in telomeres, present in both precancerous lesions and normal cells, impact cancer-specific telomere length. Reduced expression of telomerase-associated proteins hinders their suitability as ideal prognostic markers. Studies have demonstrated a significant association between telomere-related genes (TRGs) and prognosis of patients with PRAD [19, 20]. We investigated this correlation by analyzing extensive gene expression profiles in patients with PRAD and developed a predictive risk model based on TRGs. This model aims to establish a precise and personalized strategy for managing PRAD. Additionally, we assessed the clinical significance and immunotherapeutic capacity of the TRGs-based risk model and essential genes to facilitate the development of more accurate and individualized treatment approaches for patients with PRAD.

## Materials and methods

### Data acquisition and preprocessing

**Fig. 1:**
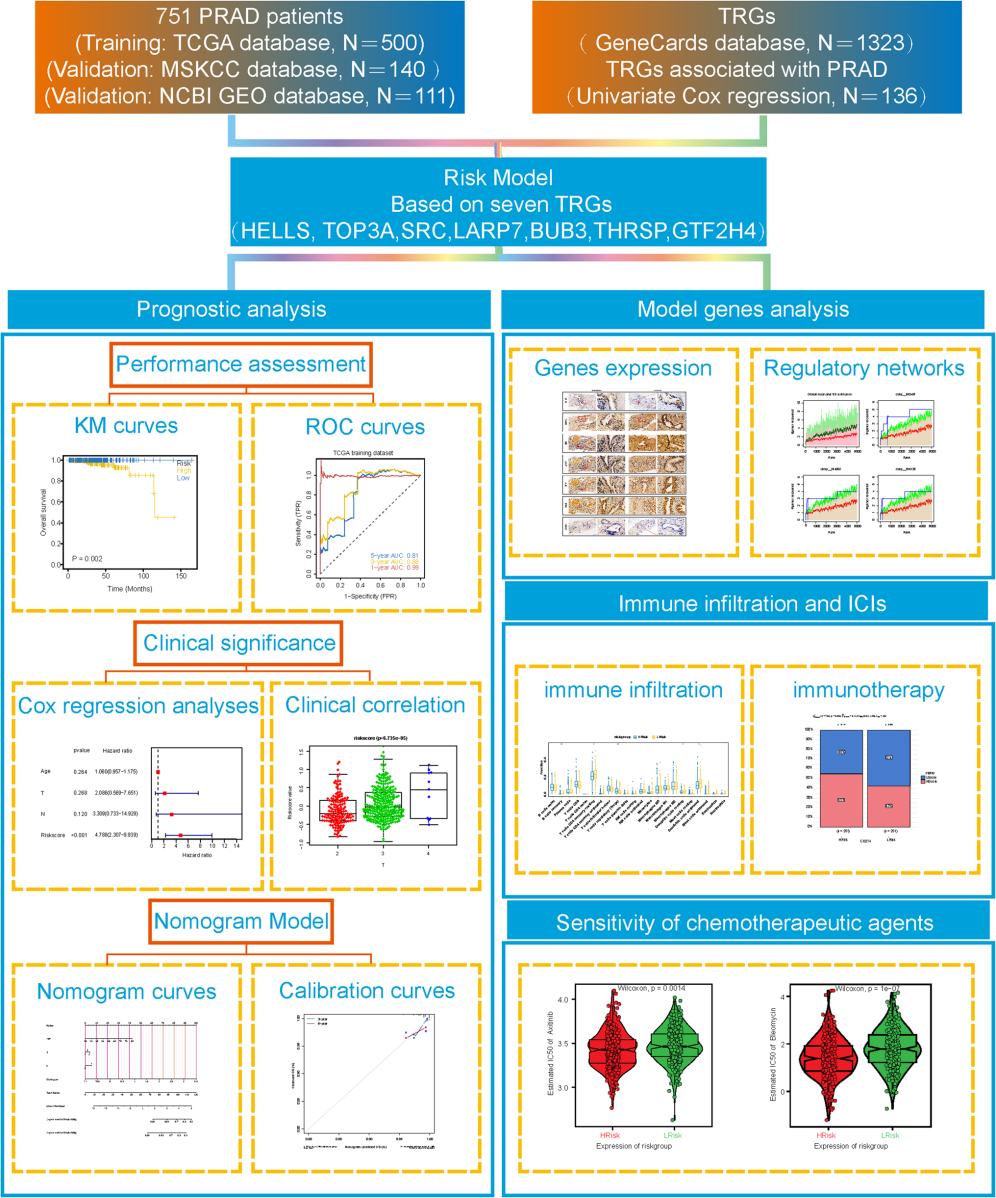
The overview design in this study

Clinical information and genomic data from 502 PRAD tumor samples and 52 standard prostate samples were downloaded from the Cancer Genome Atlas (TCGA) database (https://tcga-data.nci.nih.gov/tcga/) [18]. Transcriptomic data and survival information for 140 patients with PRAD were downloaded from the Memorial Sloan Kettering Cancer Centre project (MSKCC) using cBioPortal (http://www.cbioportal.org/). We obtained the GSE70768 dataset from the NCBI GEO (http://www.ncbi.nlm.nih.gov/geo) public database [21]. This dataset contained data from 111 patients with PRAD, including their complete expression profiles and survival information. The platform number for the annotations in the matrix file was GPL10558. Traditional screening methods were used for genetic screening [22, 23]. We obtained 1,323 TRGs (relevance score > 1) from the GeneCards database (https://www.genecards.org). All of our methods are implemented and designed to be processed in strict accordance with the operating procedures provided by the public data provider. We adhere to these guidelines to ensure the integrity and reliability of our work. All of the data were available for free online. Finally, A schematic overview of the study design with respect to the above data is shown in Fig. 1.

### Genes function enrichment analysis

Gene Set Enrichment Analysis (GSEA) was performed using the “clusterProfiler” package in R [24]. Gene ontology (GO) and Kyoto Encyclopedia of Genes and Genomes (KEGG) pathway analyses were used for the evaluation of relevant category functions. GO terms and KEGG pathways with *p* < 0.05 were considered significantly enriched. In addition, we performed functional annotation of key genomes through the Metascape database. Specific genes then underwent GO and KEGG pathway analyses. Min overlap ≥ 3 and *p* ≤ 0.01 were considered statistically significant.

### Model development and prognosis

We developed a prognostic model comprising seven genes by employing LASSO regression and multivariate Cox regression of prognosis-associated TRGs using the ‘glmnet’ package in R. After including the expression values of each gene, a risk score formula was constructed for each patient. The formula was weighted with the estimated regression coefficients of each gene using LASSO regression analysis. Based on the median risk score value, we categorized the patients into low- and high-risk groups [25]. Kaplan–Meier and log-rank statistical methods were used to compare the difference in survival between the two groups. Additionally, we determined the prognostic accuracy of the risk score model using LASSO regression analysis, stratified analysis, and receiver operating characteristic (ROC) analysis.

### Nomogram construction

We established a prognostic nomogram using regression analysis. To express the interrelationship between the variables in the risk model, we constructed line segments with scales on the same plane based on the level of gene expression and clinical symptoms at a specific ratio. By constructing a regression model, we assigned a score to each value level of each influencing factor based on their contribution to the outcome variable (as indicated by the magnitude of the regression coefficient). Subsequently, we added up the individual scores to obtain a total score, which allowed us to determine the predictive value.

### Drug sensitivity evaluation

Based on the Genomics Database of Cancer Drug Sensitivity In Cancer (GDSC, https://www.cancerrxgene.org/), the largest pharmacogenomic database available, we predicted the chemotherapeutic sensitivity of each tumor sample using the “pRRophetic” R package. We used regression to derive IC50 values for each unique chemotherapeutic drug. Additionally, we conducted 10 cross-validations on the GDSC training set to assess the accuracy of regression and prediction. All parameters, including “combats,” were set to their default levels to eliminate batch effects and calculate the mean of duplicate gene expression.

### Immune cell infiltration analysis

We analyzed RNA-seq data from various subgroups of patients with PRAD using the CIBERSORT algorithm and determined the proportions of 22 immune infiltrating cells. A *p*-value < 0.05 was considered significant for gene expression and immune cell content in the Pearson correlation analyses [26].

### Immunohistochemical (IHC) staining

Clinical tissues from patients with PRAD were fixed and sectioned. The sections were heated, dewaxed, immersed in 100% xylene, and dehydrated using a graded alcohol series. Hydrogen peroxide (0.3%) was added. And the sections were then boiled in citrate buffer (pH 6.0) for 30 min using an autoclave. Sealing was achieved by adding 10% bovine serum. Subsequently, 100 µL of primary antibody, diluted in 1% bovine serum albumin, was added to each section. The sections were then incubated overnight in a humidified chamber at 4 °C with the following primary antibodies: HELLS antibodies (1:300, PA5-64099, Thermo Fisher), TOP3A antibodies (1:200, PA5-121538, Thermo Fisher), SRC antibodies (1:200, ab109381, abcam), LARP7 antibodies (1:200, 17067-1-AP, proteintach), THRSP antibodies (1:400, 13054–1-AP, proteintach), GTF2H4 antibodies (1:200, PA5-103346, Thermo Fisher), and BUB3 (1:200, ab133699, abcam). The corresponding secondary antibodies were added to tissue sections. The sections were observed under a microscope after 3,3′-diaminobenzidine development and hematoxylin staining. The study received approval from the Ethics Committee of the Fifth Hospital of Guangzhou Medical University, specifically for the utilization of diagnostic residual tissue for research purposes (Approval No: KY01-2020-03-01). Prior to their participation in the study, all individuals involved provided their consent through the signing of an informed consent form. The rights of all participants were upheld, including the assurance of confidentiality and the freedom to withdraw from the study at any point. The execution of the experiment adhered strictly to the relevant guidelines and regulations.

### Gene set variance analysis (GSVA)

To identify differentially expressed genes between the high- and low-risk groups, we performed an enrichment analysis of the gene set using the PRAD expression profile (GSEA; http://www.broadinstitute.org/gsea) [27]. The “c2.cp. Kegg.v7.4.entrez.gmt” gene set was downloaded from the MSigDB database and applied filters to include gene sets with sizes ranging from 15 to 500 genes. GSEA was then performed using the “clusterProfiler” package in R. GSVA was performed using the GSVA package. The “limma” algorithm was used to determine pathway differences between the high- and low-risk groups. By intersecting the results obtained from the transcriptome-based GSEA and proteome-based GSVA, we identified differential pathways between the high- and low-risk groups.

### Regulatory network analysis

Transcription factors are proteins that bind to specific DNA sequences. The R package “RcisTarget” was used to predict the transcription factors associated with the model genes. The area under the curve (AUC) was calculated for each motif-motif set pair to evaluate the overexpression of each motif within a gene set. Subsequently, we calculated the normalized enrichment score (NES) for each motif based on the distribution of AUCs for all motifs in the genome.

### Statistical analysis

All statistical analyses were performed using the R software (version 4.0.2). Survival curves were generated using the Kaplan–Meier method, and the differences between the curves were assessed using the log-rank test. Furthermore, a multivariate analysis was conducted using the Cox proportional hazards model. Both single- and multi-factor Cox analyses were performed by using the R survival package, and forest plots were generated to visualize the results. Two-group comparisons were assessed using the Wilcoxon test function, whereas multi-group comparisons were assessed using the Kruskal–Wallis test function. Correlation analysis was performed using the Pearson method. The ssGSEA scores for the samples were calculated using the ssGSEA function in the “GSVA” package. *P* < 0.05 was considered significant.

## Results

### Screening and functional enrichment analysis of TRGs

**Fig. 2:**
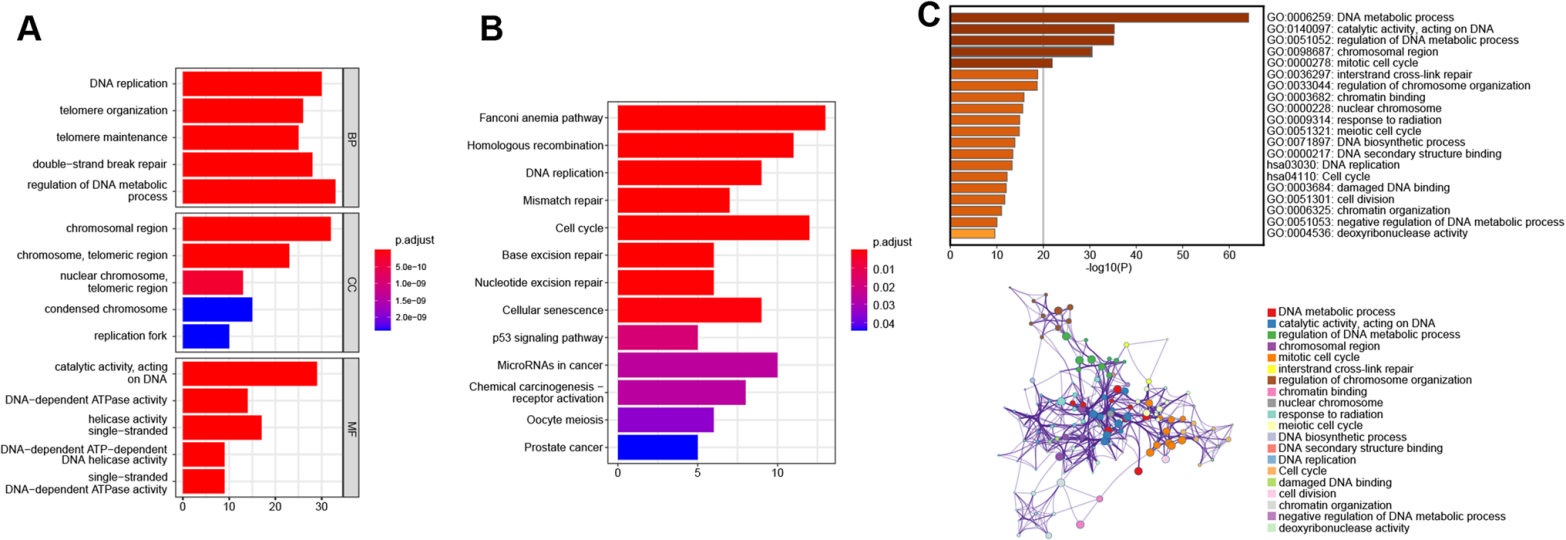
Enrichment analysis of TRGs associated with PRAD prognosis. **A**,** B** Gene ontology (GO) and Kyoto Encyclopedia of Genes and Genomes (KEGG) pathway enrichment analysis using the “Clusterprofiler” R package. **C** Metascape-based GO-KEGG pathway enrichment analysis. The lower panel shows the cluster network composed of enriched pathways, where nodes sharing the same cluster are usually close to each other

The univariate Cox regression analysis revealed 136 TRGs associated with PRAD prognosis (*p* < 0.05) (Additional file 1: Supplementary File 1). We also performed a functional enrichment analysis of these TRGs. The GO enrichment analysis revealed that the majority of these genes were related to telomere regulation, chromosomal area, DNA-dependent ATPase activity, and other related pathways (Fig. 2A). In addition, the KEGG enrichment analysis revealed that these genes were considerably enriched in pathways such as homologous recombination and cell cycle (Fig. 2B). Furthermore, the Metascape enrichment analysis revealed that these genes were predominantly involved in biological activities such as DNA metabolism, catalytic activity, DNA activity, and DNA metabolism (Fig. 2C). To investigate the potential relationships between the TRGs, we performed a Protein–protein interaction (PPI) network analysis using the Cytoscape software (Additional file 1: Supplementary Fig. S1). The results imply that the interactions between the TRGs are complex, closely related, and closely associated with DNA-related biological processes, suggesting that 136 TRGs may play an essential role in the progression and prognosis of PRAD.

### Construction and validation of the TRG-based risk model

**Fig. 3:**
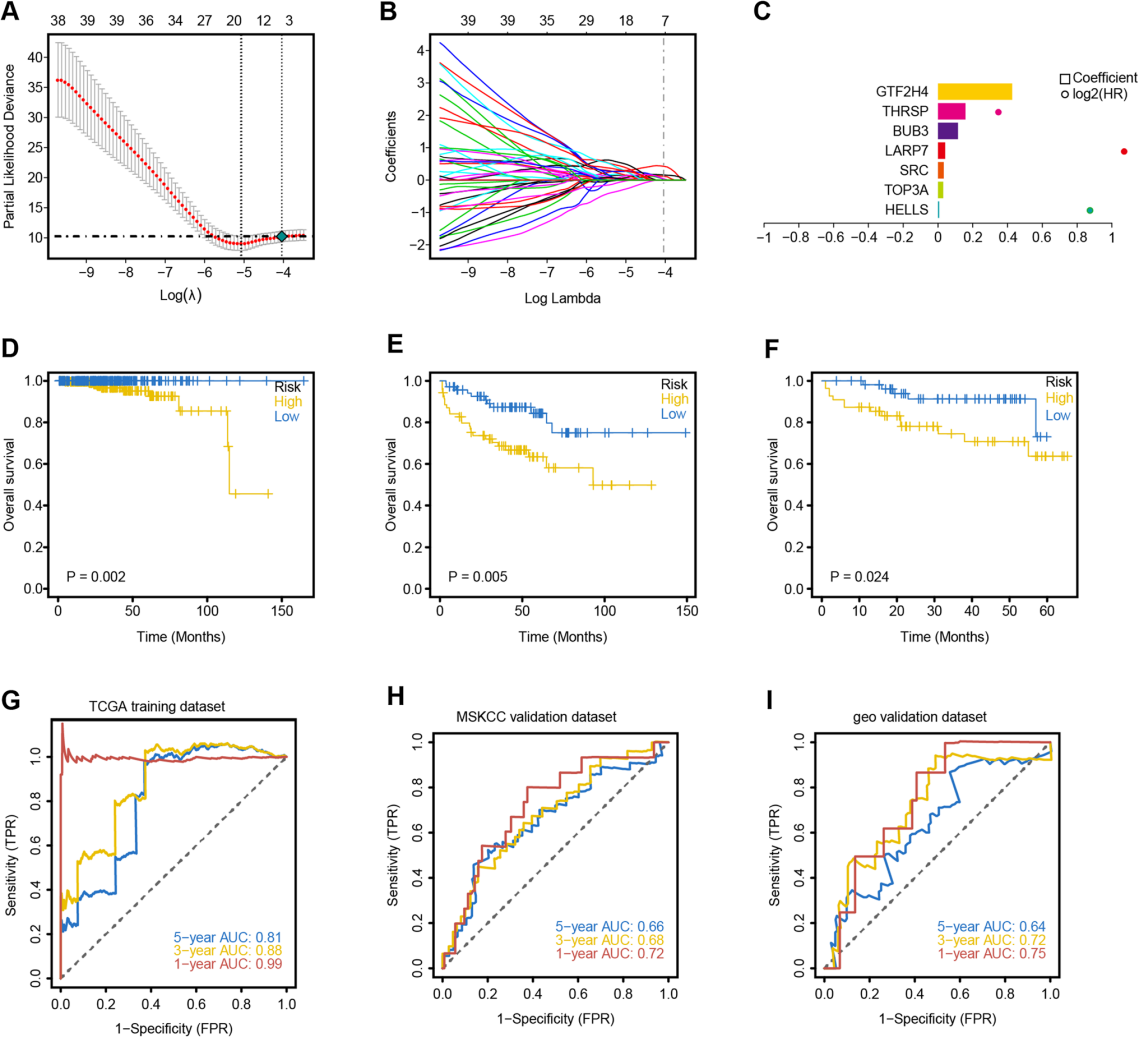
The risk model was constructed in the Cancer Genome Atlas-Prostate Adenocarcinoma (TCGA-PRAD) cohort and validated in the Memorial Sloan-Kettering Cancer (MSKCC) and Gene Expression Omnibus (GEO) cohorts. **A** Ten-fold cross-validation was used to adjust the parameter selection for the LASSO model to determine the minimum λ value. **B** Distribution of LASSO coefficients for TRGs and gene combinations with minimum λ values. **C** Seven model genes (*GTF2H4*, *THRSP*, *BUB3*, *LARP7*, *SRC*, *TOP3A*, and *HELLS*) were selected using the LASSO regression algorithm. **D–F** Survival curves were constructed based on the model genes in the TCGA-PRAD (*p* = 0.002), MSKCC (*p* < 0.005), and GEO (*p* = 0.024) cohorts. **G–I** Receiver operating characteristic (ROC) analysis

We used 136 TRGs as candidate genes for modeling and selected TCGA-PRAD cohorts with complete expression profiles and survival data as the training set. Detailed clinical characteristics of the 500 patients with PRAD in the TCGA-PRAD cohort are shown in Additional file 1: Supplementary Table 1. The MSKCC and GSE70768 patient cohorts were used as external validation sets. Additionally, clinical data were collected from patients with PRAD, and the final candidate genes were screened using the LASSO regression algorithm (Fig. 3A–C). The formula for the risk model was: Risk Score = (*HELLS* × 0.00716375741617321) + (*TOP3A* × 0.0301970796315794) + (*SRC* × 0.0327209383443109) + (*LARP7* × 0.0408035537429572) + (*BUB3* × 0.11506165146503) + (*THRSP* × 0.158045396171819) + (*GTF2H4* × 0.426874220611804). The patients with PRAD were divided into high- and low-risk groups based on the median risk score value. In the training cohort, the high-risk group had significantly lower OS than the low-risk group (Fig. 3D). Similarly, we validated the stability of the risk model by calculating survival differences between patients with PRAD in the GEO and MSKCC cohorts (Fig. 3E, F). To further assess the performance of the risk model, we generated time-dependent ROC curves. The risk model’s 1-, 3-, and 5-year survival AUC values were 0.99, 0.88, and 0.81, respectively (Fig. 3G). We also performed ROC curve analysis on external validation sets to validate the accuracy of the model. The results indicated that the risk model can be useful in predicting prognosis (Fig. 3H, I).

### Differential expression and regulatory networks of the model genes

**Fig. 4:**
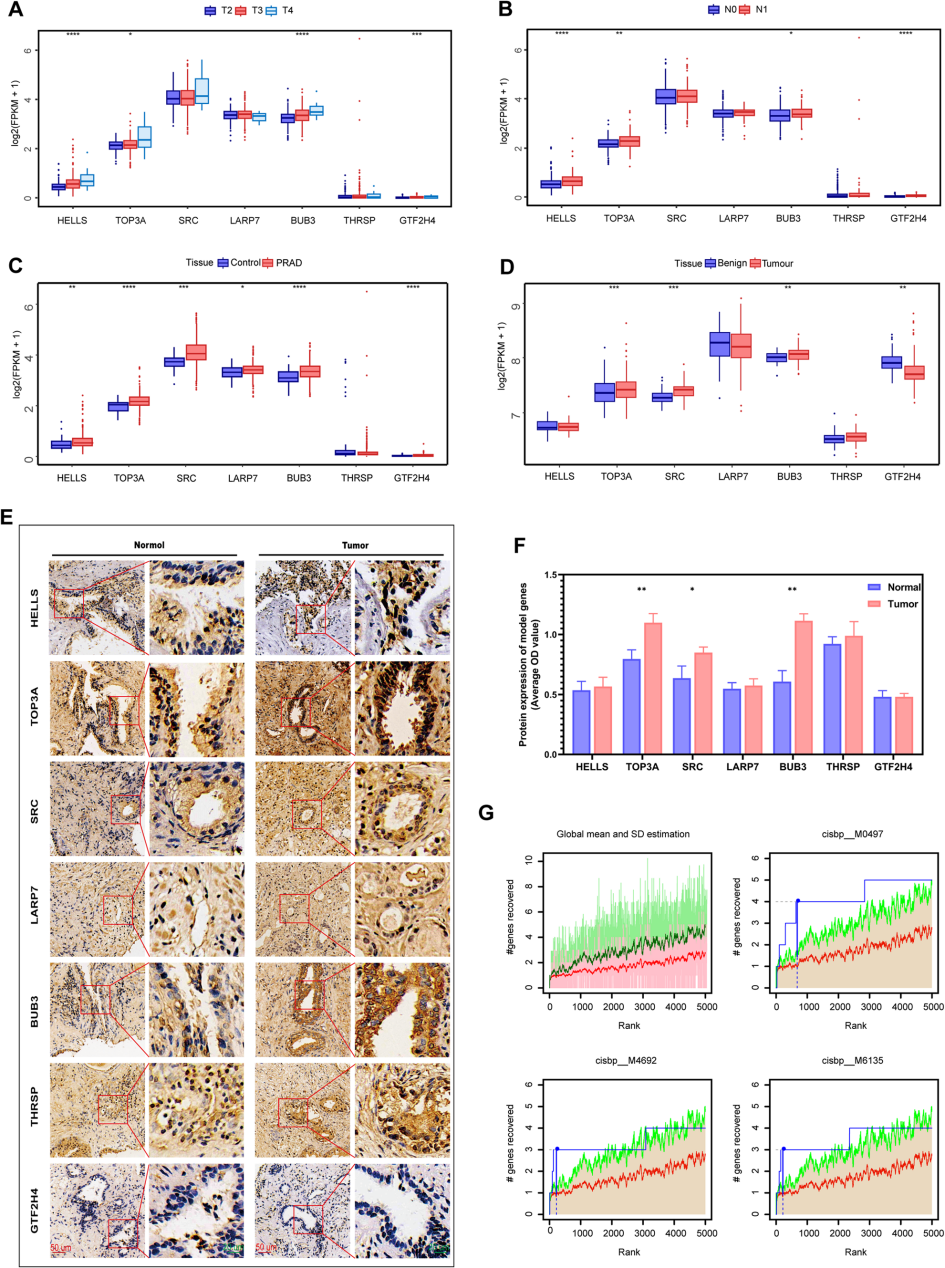
Expression differences and specific regulatory mechanisms of the model genes. **A** Differential expression levels of the model genes at different clinical T stages (T2, T3, and T4). **B** Differential expression levels of the model genes at different clinical N stages (N0 and N1). **C, D** Comparison of mRNA expression levels for the critical genes between PRAD and normal tissue in TCGA and GEO cohorts. The seven critical genes used to construct the risk model are represented on the horizontal axis, and the gene expression levels calculated using log2 (FPKM + 1) are represented on the vertical axis. *p < 0.05, **p < 0.01, ***p < 0.001, and ****p < 0.0001. **E** Representative immunohistochemical images of the seven model genes in PRAD tissues and adjacent normal tissues (n = 3). Scale bars, 100 μm (left) and 20 μm (right). **F** Statistical analysis of the average OD value based on stained areas. **G** Cumulative recovery curves for four base sequences with high AUC values. The red line represents the mean of the recovery curve for each motif, while the green line represents the mean ± standard deviation (SD). The blue line represents the recovery curve of the current motif. The maximum enrichment level is the maximum distance point (mean + SD) between the current motif and the green curve

We performed a correlation analysis between clinical data and model genes and found that *HELLS*, *TOP3A*, *BUB3*, and *GTF2H4* were significantly positively correlated with clinical T and N stages of PRAD (Fig. 4A, B). To evaluate the expression levels of the seven critical genes used to construct the risk model in PRAD and normal tissues, we downloaded RNA sequencing data from TCGA and GEO databases for differential analysis. Our findings revealed that the expression levels of *TOP3A*, *SRC*, and *BUB3* were significantly elevated in the tumor groups of both the TCGA and GEO cohorts. Conversely, *HELLS* and *LARP7* demonstrated high expression exclusively in the TCGA cohort (Fig. 4C, D). To further investigate the expression of the model genes in clinical samples, we collected tissues from patients with PRAD and performed IHC staining on cancerous and adjacent normal tissues. The IHC representative results revealed that TOP3A, SRC and BUB3 exhibited stronger staining in PRAD tissues compared to adjacent normal tissues (Fig. 4E, F). The staining intensity of other genes did not show significant differences between cancerous and adjacent normal tissues.

Next, we investigated the specific regulatory mechanisms of the model genes in the risk model. We identified several transcription factors and common regulatory mechanisms associated with these genes. Using cumulative recovery curves, we conducted an enrichment analysis of these transcription factors (Fig. 4G). The results show that the motif annotation with the highest NES was cisbp_M0497. This motif was overrepresented in the *BUB3*, *GTF2H4*, *LARP7*, and *TOP3A* genes (Additional file 1: Supplementary Fig. S2).

### Exploration of the prognostic value and clinical relevance of the risk model

**Fig. 5:**
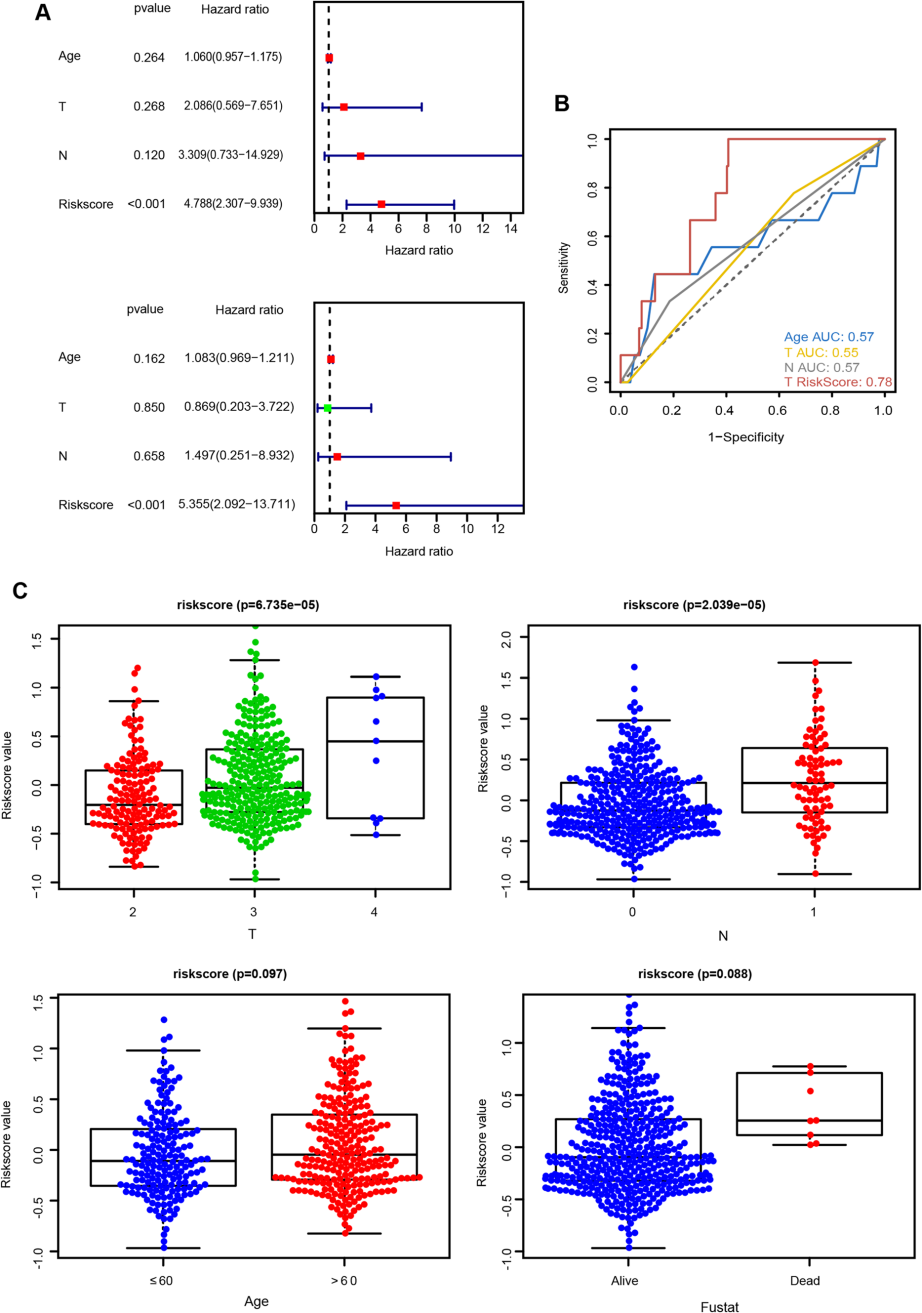
The prognostic value and clinical relevance of the risk model. **A** Single- and multi-factor regression forest plots. Red indicates risk factors, and green indicates protective factors. **B** Comparison of the ROC curve and area under the curve (AUC) values between the risk score and other clinical factors. **C** Clinical correlation between risk score and PRAD. Differences between risk score and T, N, age, and Fustat (*p* < 0.05 was considered statistically significant)

We validated the prognostic value of the risk model in each patient with PRAD. The univariate and multivariate analyses revealed that the risk score was also an independent prognostic factor for PRAD (Fig. 5A). Additionally, the AUC value of the risk score was greater than that of the conventional prognostic scoring system (AUC: risk score = 0.78), further indicating that the risk model has superior prognostic value over other clinical characteristics (Fig. 5B). To investigate further the correlation between the risk model and clinical indicators, we presented the risk score values of all samples as box plots (Fig. 5C). We discovered that the risk score values were significantly correlated between T and N clinical groups but not between age and survival status groups.

### Construction and validation of the risk score-related nomogram model

**Fig. 6:**
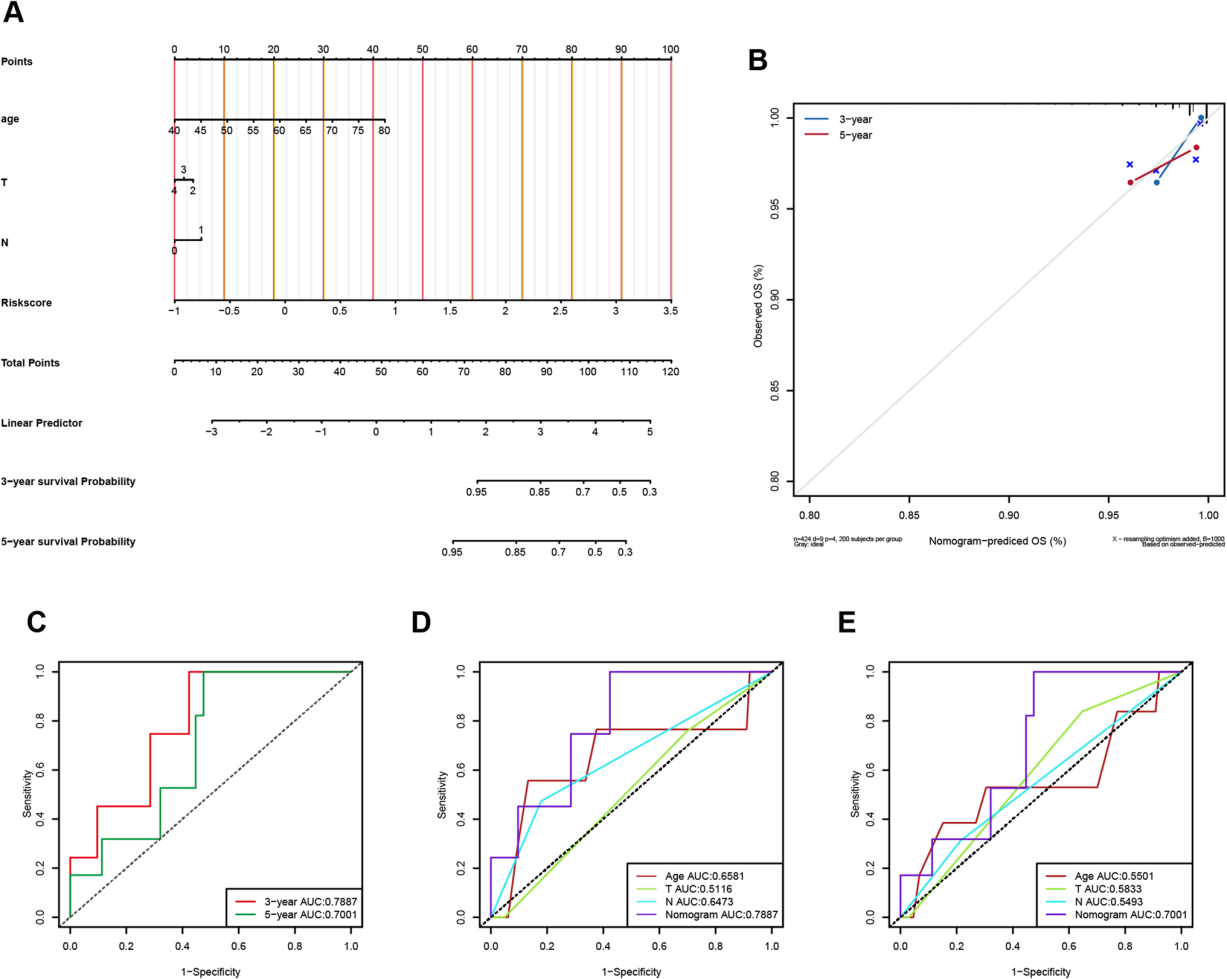
Construction of the risk score-related nomogram model. **A** The nomogram model was constructed using risk scores and other clinical characteristics. **B** 3- and 5-year nomogram calibration curves for OS. **C** ROC curves for predicting 3- and 5-year survival. **D** Comparison of the ROC curve and AUC values of 3-year survival between the nomogram and other clinical factors. **E** Comparison of the ROC curve and AUC values of 5-year survival between the nomogram and other clinical factors

To further improve the predictive power and applicability of the risk model, a predictive nomogram (Fig. 6A) was developed by integrating the age, T category, N category, and risk score of patients with PRAD. The nomogram can systematically predict the 3- and 5-year overall survival (OS) of patients with PRAD. The calibration plots demonstrated that the nomogram model had high accuracy and congruence between actual and predicted results (Fig. 6B). The 3- and 5-year survival AUC values of the predictive nomogram were 0.78 and 0.70, respectively (Fig. 6C). Comparing the 3-and 5-year survival AUC values of the nomogram with other clinical factors, we found that the nomogram had a substantially higher AUC value, indicating that our nomogram has good discrimination (Fig. 6D, E). The decision curve analysis indicated that the nomogram has greater clinical applicability (Additional file 1: Supplementary Fig. S3). These findings suggest that the nomogram model is suitable for predicting the 3-year and 5-year survival of patients with PRAD.

### Exploration of signaling mechanisms related to the risk model

**Fig. 7:**
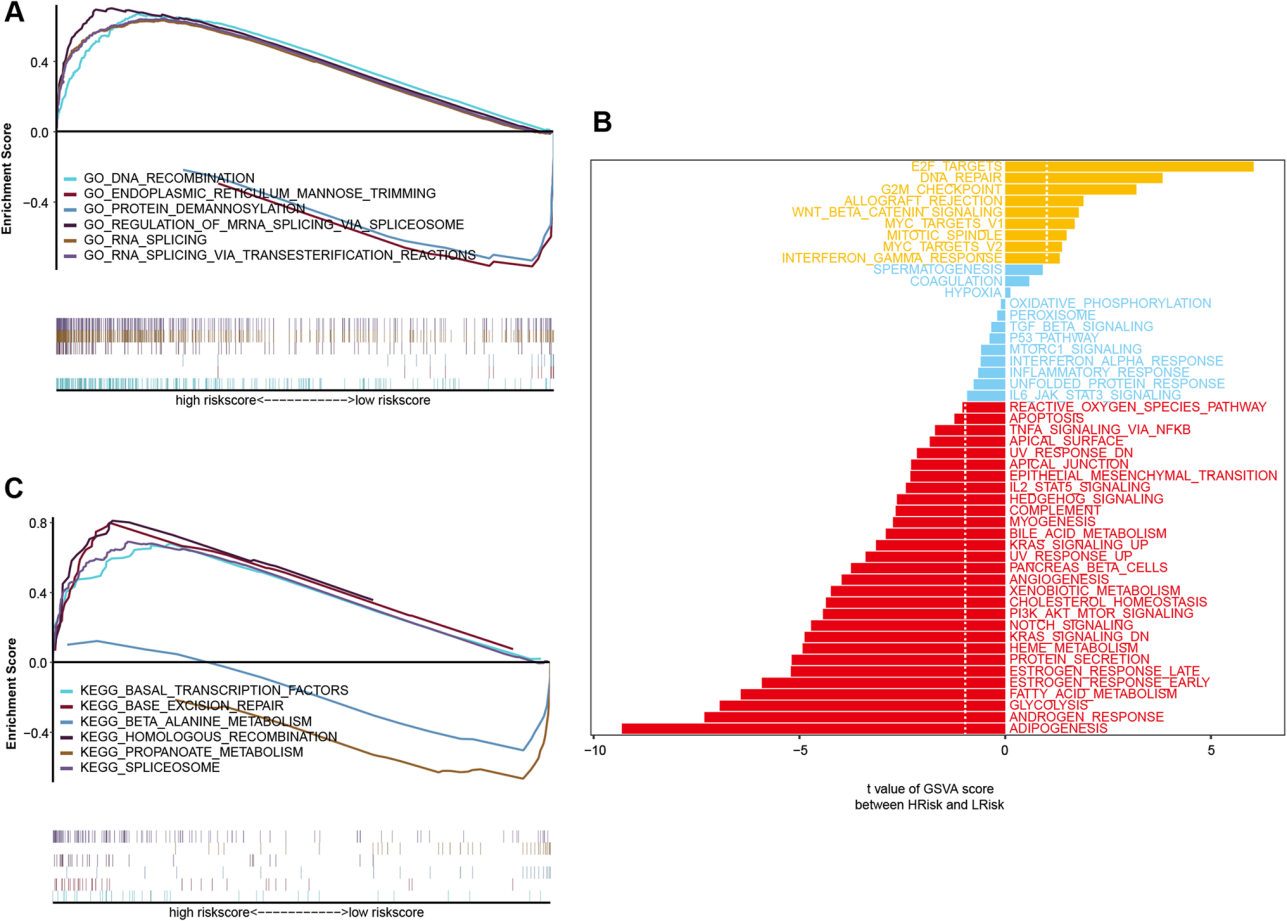
Differences in signaling pathways between the high- and low-risk groups. **A**, **B** GO-KEGG pathways enriched in high- and low-risk groups. **C** Pathway activity was scored using gene set variance analysis (GSVA), with Hallmark as the background gene set

To elucidate the potential impact of the risk score on the molecular progression of PRAD, we analyzed the distinct signaling pathways implicated in the high- and low-risk groups. The GSEA analysis revealed significant enrichment in several related pathways, such as the GO enrichment pathways of DNA recombination and endoplasmic reticulum mannose trimming (Fig. 7A), as well as the KEGG enrichment pathways of fundamental basal transcription factors and base excision repair (Fig. 7B). Additionally, the GSVA analysis indicated that differential pathways in both groups were predominantly enriched in E2F targets, DNA repair, G2M checkpoint, and other signaling pathways, suggesting that alterations in these signaling pathways in the high- and low-risk groups affected prognosis (Fig. 7C). Our findings suggest that these signaling pathways are potential therapeutic targets for developing more effective treatments for high-risk patients with PRAD.

### Analysis of the tumor immune microenvironment in the high- and low-risk groups

**Fig. 8:**
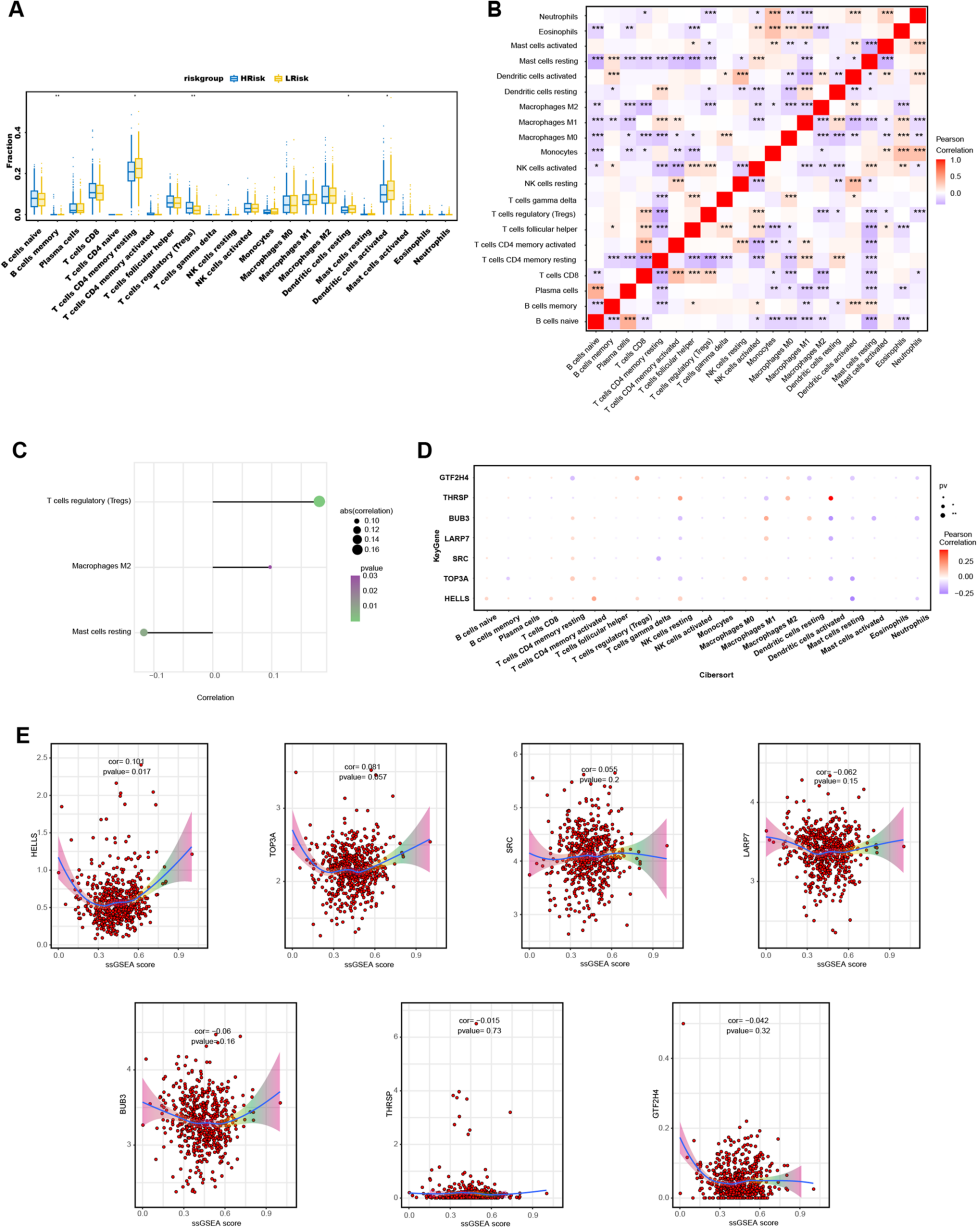
Analysis of tumor microenvironment and immune correlation. **A** Differences in immune cell proportion between patients in the high- and low-risk groups. Blue indicates patients in the low-risk group, and yellow indicates patients in the high-risk group (*p < 0.05, **p < 0.01, and ***p < 0.001). **B** Correlation between risk score and immune cells. **C** Pearson's correlation among 22 high- and low-risk immune cell types. **D** Correlation between seven model genes and 21 immune cell types. **E** Correlation between immune ssGSEA scores and model gene expression

The tumor immune microenvironment considerably impacts tumor diagnosis, survival outcomes, and clinical treatment sensitivity[28]**.** Consequently, we analyzed the relationship between risk score and tumor immune infiltration (Additional file 1: Supplementary Fig. S4). The immune cell content of each patient with PRAD is shown in Fig. 8A. The results revealed that the low-risk group samples had significantly higher levels of T-cell CD4 memory quiescent, dendritic cell (DC) quiescent, and mast cell quiescent and significantly lower levels of regulatory T cells (Tregs) compared to the high-risk group. Additionally, we performed a Pearson correlation analysis on 22 immune cells, revealing multiple pairs of significant correlations between several immune cells and risk scores (Fig. 8B). Furthermore, risk scores were positively correlated with Tregs and macrophage M2 but negatively correlated with mast cell resting (Fig. 8C). We also investigated the correlation between the seven model genes and the immune cells (Fig. 8D). The expression levels of the model genes showed significant correlations with various immune cells. For example, *BUB3* was positively correlated with T cell CD4 memory quiescence, macrophage M1, and DC quiescence but negatively correlated with natural killer cell quiescence, DC activated, mast cell activated, and neutrophils. Additionally, we performed a correlation analysis between these model genes and immune ssGSEA scores (the set of immune-related genes with correlation scores > 7 in the GeneCards database) (Fig. 8E) and found a positive correlation between *HELLS* expression and immune scores.

### Correlation between risk score and immune-related genes

**Fig. 9:**
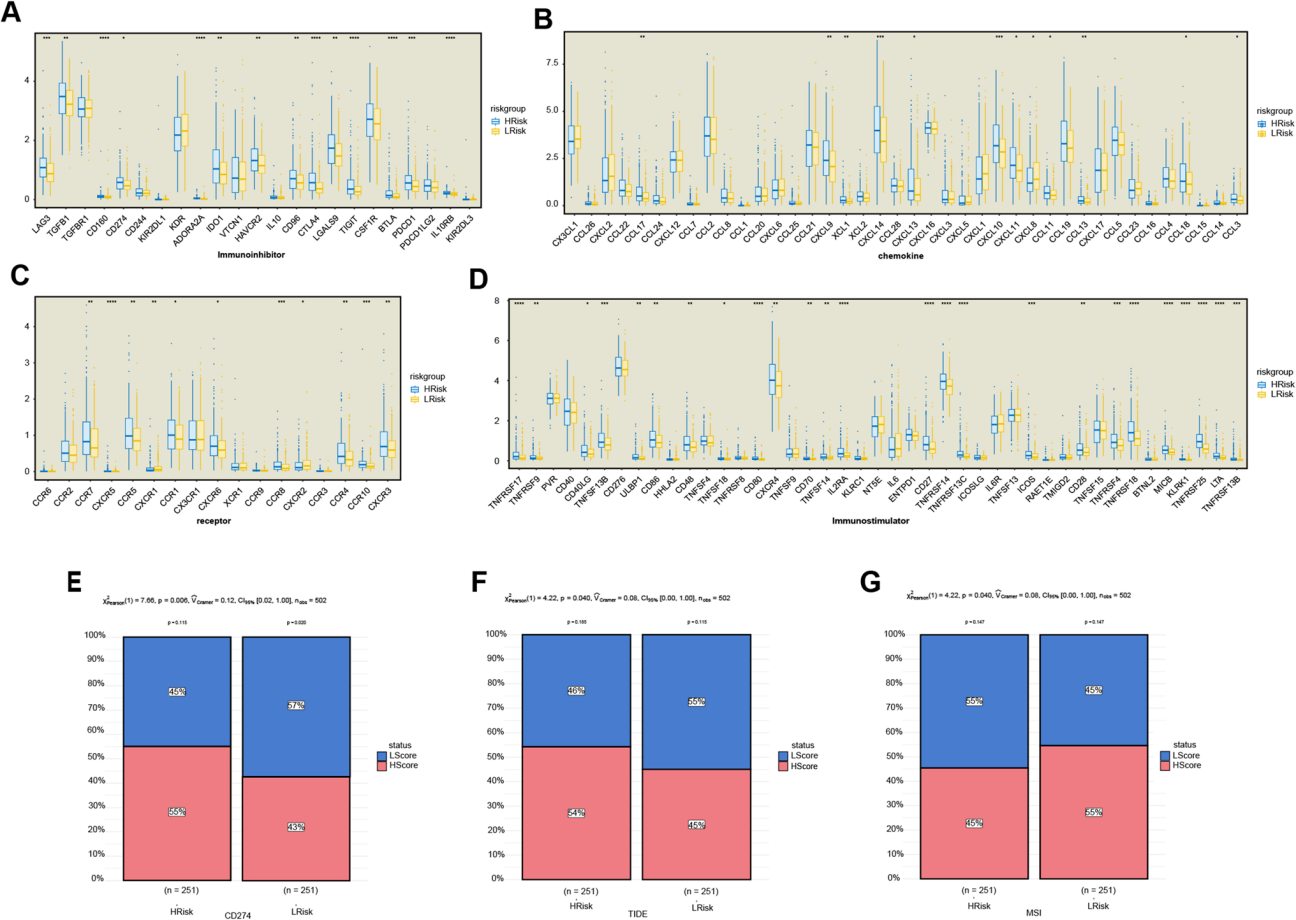
Correlation between risk score and immune-related genes. **A**–**D** Differences in the expression of immune-related genes in samples from high- and low-risk groups (*p < 0.05, **p < 0.01, and ***p < 0.001). **E**–**G** Tumor Immune Dysfunction and Exclusion (TIDE) estimation showing *CD274*, microsatellite instability (MSI), and TIDE scores. Abbreviations: *CD274*, the cluster of differentiation 274, also called PD-L1

Immune checkpoint inhibitors (ICIs) have been used in recent years for the treatment of several cancers. We explored the differences in the effectiveness of treatment with ICIs between the high- and low-risk groups to inform the clinical management of PRAD. We extracted several sets of immune-related genes, including immunomodulators, from the TISIDB database to further investigate the association between risk scores and immune checkpoints, immunomodulators, chemokines, and cell receptors (Fig. 9A–D)(Additional file 1: Supplementary Fig. S5). We found that nearly all immune checkpoint genes (*CTLA-4*, *TGFB1*, *CD274*, *LAG-3*, *TIGIT*, and *BTLA*) were considerably expressed in the high-risk group relative to the low-risk group, which may partly explain the favorable prognosis associated with the low-risk group and suggest that patients with high risks may benefit from treatment with ICIs. In addition, we analyzed the effect of immunotherapy in the high- and low-risk groups using the tumor immune dysfunction and exclusion (TIDE) algorithm. Significant differences in *CD274*, microsatellite instability (MSI), and TIDE scores were observed between the high- and low-risk groups (Fig. 9E–G). The higher expression of *CD274* and TIDE scores and lower MSI scores in the high-risk group further indicated a poor prognosis. However, although the effectiveness of ICIs is unknown, high-risk patients may benefit more than low-risk patients.

### Sensitivity of chemotherapeutic agents in PRAD

**Fig. 10:**
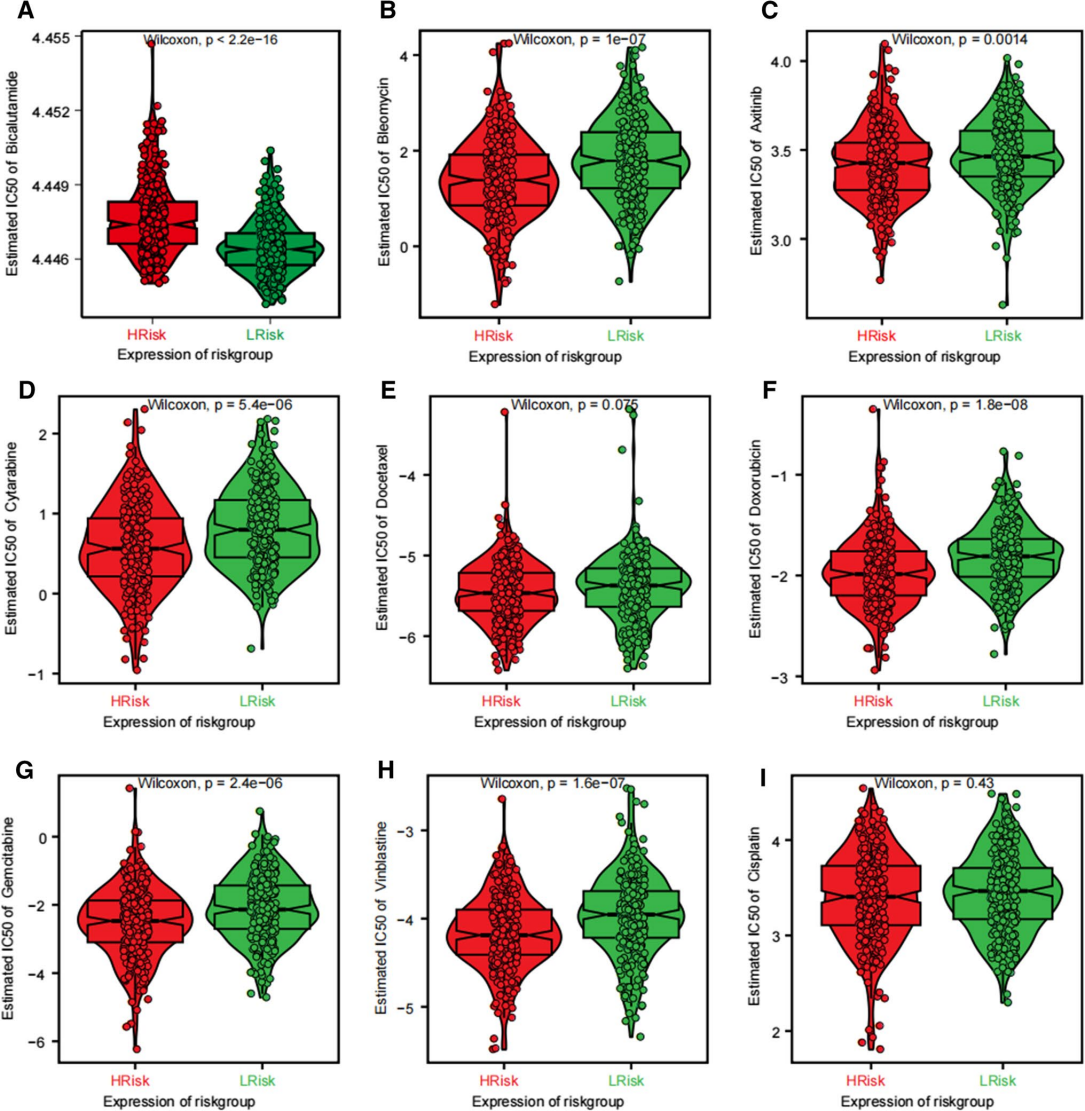
Correlation of risk score and sensitivity of common chemotherapeutic agents

In the clinical management of PRAD, the use of novel endocrine drugs has become increasingly prevalent. Numerous studies have demonstrated that patients who exhibit sensitivity to these new endocrine therapies tend to have significantly extended survival rates. In this study, we focus on bicalutamide, a prominent agent in this category [29]. Our findings indicate that the low-risk group exhibits a higher sensitivity to bicalutamide compared to the high-risk group (Fig. 10A). This observation further substantiates the superior clinical prognosis associated with the low-risk group. In addition, several studies have demonstrated that conventional endocrine therapy does not improve OS in patients with PRAD. However, early administration of neoadjuvant chemotherapy combined with endocrine therapy can significantly improve survival [30]. For example, treatment regimens with docetaxel combined with endocrine therapy have yielded definitive clinical results and are recommended as part of the standard of care for men who are physically strong and beginning long-term hormone therapy. Using the “pRRophetic” R package, we predicted the chemotherapy sensitivity of each tumor sample, offering additional drug options and potential regimens for neoadjuvant chemotherapy. The results revealed a significant correlation between risk scores and drug sensitivity to gemcitabine, doxorubicin, axitinib, bleomycin, cytarabine, doxorubicin, and vincristine but not to cisplatin (Fig. 10B–I).

## Discussion

Telomeres play a crucial role in the development of PRAD. In the early stages of PRAD, there is a noticeable shortening in the length of telomeres. This shortening of telomeres leads to genomic instability, resulting in the inactivation of tumor-suppressor genes and the production of oncogenes involved in the initiation and progression of PRAD [31]. The length of telomeres in PRAD tissues can be a potential prognostic marker [32]. PRAD cells activate telomerase to maintain the shortened telomere length at a level that supports unlimited replication. In contrast, normal prostate cells have undetectable levels of telomerase activity. Telomere stability is closely related to the prognosis of PRAD. In castration-resistant PRAD, telomere dysfunction leads to more invasive cancer cells [33]. Studies have demonstrated a significant association between certain TRGs affecting telomere alterations and PRAD prognosis. To investigate the predictive value of TRGs, we constructed a risk model based on TRGs using a public database. Subsequently, we validated the predictive ability and clinical relevance of this risk model. Through our study, we aim to offer novel insights that may contribute to identifying potential treatment options for PRAD.

We used LASSO regression and Cox survival analyses to construct a risk model based on seven TRGs: *HELLS*, *SRC*, *LARP7*, *BUB3*, *THRSP,* and *GTF2H4*. We found that *HELLS*, *TOP3A*, *BUB3* and *GTF2H4* showed significant correlations with PRAD’s clinical T and N stages. Furthermore, immunohistochemical analysis of clinical PRAD tissues demonstrated elevated staining levels of *TOP3A*, *SRC*, and *BUB3* compared to adjacent normal tissues. TOP3A is an enzyme localized in the nucleus and mitochondria. It can alter DNA topology by creating temporary breaks in the DNA backbone [34]. Notably, TOP3A dysfunction may affect the stability of the nuclear or mitochondrial genome [35]. SRC is an intracellular non-receptor tyrosine kinase that plays a crucial role in regulating various biological processes associated with tumor proliferation, migration, invasion, and angiogenesis [36] Studies have shown aberrant activation or expression of SRC kinases in various tumor tissues, including prostate, breast, lung, and colorectal cancers [37–39]. BUB3 is a mitotic checkpoint protein that regulates the spindle assembly checkpoint and plays an essential role in cell division [40]. Studies have demonstrated significant upregulation of BUB3 mRNA expression in prostate cancer compared to benign prostatic hyperplasia. Furthermore, increased BUB3 upregulation was found to be associated with FOXA1 expression levels in PRAD, and patients with PRAD who had high BUB3 expression tended to show an unfavorable prognosis [41]. Further investigations are required to delve the specific mechanisms of these three genes in prostate cancer.

Notably, the risk model exhibited robust predictive capabilities, as evidenced by OS and ROC curve analyses. The risk model exhibited powerful predictive capabilities, as evidenced by overall survival OS and ROC curve analyses. Additionally, it proves to be an independent prognostic factor for patients with PRAD and has shown superiority compared to traditional predictive scoring systems. We found a significant correlation between the risk model and the T/N tumor staging. Through GSEA and GSVA, we identified pathways enriched in both the high- and low-risk groups, including DNA recombination, endoplasmic reticulum mannose modification, fundamental transcription factors, and base excision repair pathways. However, there were differences in specific ways, such as DNA repair and the G2M checkpoint. These differential signaling pathways may be potential therapeutic targets that could lead to the development of more effective treatments for high-risk patients with PRAD. Moreover, our integrated nomogram demonstrated high accuracy in predicting the 3- and 5-year survival rates of patients with PRAD. Although risk models have shown excellent predictive power in patients with PRAD, comparing our risk model with other published prognostic models is essential to determine its superiority. In clinical practice, surgeons typically assess prognosis and guide treatment based on the TNM stage, biopsy Gleason score, and pretreatment prostate-specific antigen levels. Therefore, to develop a more accurate nomogram, we need to collect additional samples and incorporate Gleason score staging and prostate-specific antigen levels into our model [42].

Immune cells are essential in the fight against tumours [43]. Dendritic cells (DCs) are the most potent antigen-presenting cells [44, 45]. They can overcome the local immunosuppressive zones established by tumours and present the captured antigens directly to T lymphocytes. DCs can induce cell-mediated immune responses and exert anti-tumour effects through cytotoxic T cells [46]. The number of CD4 + or CD8 + cells negatively correlates with biochemical recurrence and tumour-specific survival [47]. However, memory CD4 + T cells can exert anti-tumour immune effects through multiple mechanisms, such as directly regulating the expression of granzyme and perforin and indirectly regulating the production of CD8 + memory T cells [2]. Regulatory Tregs are essential for maintaining immune tolerance and homeostasis. Tregs can inhibit the activation of CD8 + T cells by suppressing the expansion and immunogenicity of DCs, resulting in poor immunotherapeutic outcomes in tumours [48]. In addition, Tregs can also promote the differentiation of macrophages into M2-like tumour-promoting phenotypes, which can enhance the proliferation of tumour cells [49]. In this study, we found that the samples in the low-risk group had significantly higher levels of resting memory CD4 + T cells, resting DCs, and resting mast cells than those in the high-risk group. Moreover, the risk scores were significantly and positively correlated with Tregs and M2-like macrophages. These results suggest that higher risk scores are associated with a worse prognosis in tumour patients, which is consistent with the previous research.

Li X et al. showed that TIDE scores were associated with response to immunotherapy and MSI scores with tumour prognosis [50]. High expression of *CD274,* a gene encoding the immune checkpoint protein PD-L1, was associated with evasion of immune surveillance [51]. In our study, the high expression level of *CD274*, the high TIDE score, and the low MSI score in the high-risk group suggested that this group had a worse prognosis than the low-risk group. Immunotherapy with ICIs has shown significant efficacy in treating various cancers by blocking the inhibitory signalling pathways of T cells [52, 53]. We analysed the expression levels of immune-related genes, including immunomodulators and immune checkpoints, and revealed significant differences between the high- and low-risk cohorts. High-risk patients might benefit more from ICIs, but the effect was unclear and needed further validation. Studies have shown that conventional antitumor chemotherapeutic agents, in addition to directly inhibiting tumor growth, can induce immunogenic cell death [54, 55]. There was a significant correlation between the risk scores and the sensitivity to multiple chemotherapeutic agents, with lower IC50 values in the high-risk group than in the low-risk group. The risk model developed could predict chemotherapy response and help identify the most suitable chemotherapy regimen for each PRAD patient. Combination therapies, such as ICIs plus chemotherapy, might be a promising treatment option for advanced PRAD patients with high-risk scores.

We developed the first TRGs-based PRAD risk model using the TCGA database to compare the expression levels of model genes in primary prostate cancer tissues and adjacent normal tissues. We found that the low-risk group had significantly higher levels of infiltration of resting memory CD4 + T cells, resting DCs, and resting mast cells than the high-risk group. In addition, the low-risk group had significantly lower Tregs and M2-like macrophages associated with immunosuppression and tumour progression. These findings provide a clinical approach to determine the prognosis of PRAD patients and further validate the favourable prognosis and the immune environment of the low-risk group. Although the high-risk group had a worse prognosis, they also had higher sensitivity to chemotherapeutic agents and ICIs, as indicated by the lower IC50 values and the higher expression of immune-related genes. This suggests that chemotherapy combined with ICIs might be an effective treatment option for patients in the high-risk group. These findings provide a clinical approach to determining the prognosis of patients with PRAD and an essential basis for exploring potential therapeutic options. Meanwhile, more clinical data and samples are needed to confirm the results' accuracy.

## Conclusion

In summary, we constructed a risk model based on seven TRGs to predict the prognosis of patients with PRAD. This model showed commendable performance in independently evaluating the prognosis of OS at 1, 3, and 5 years. In addition, we further determined the relationship between the immune environment, ICIs, chemotherapy sensitivity and risk models. Compared to the low-risk group, the high-risk group had a higher response to chemotherapy and immunosuppression, which provided potential guidance to treatment options for patients in the high-risk group.

### Supplementary Information

**Additional file 1. ** Telomere-related prognostic genes **Supplementary Table 1:** Detailed clinical characteristics of the 500 PRAD patients. **Supplementary Fig. S1:** PPI graph of TRPGs, PPI: Protein-protein interaction. **Supplementary Fig. S2:** Enrichment of motifs and transcription factors. **Supplementary Fig. S3:** DCA analysis curves for nomogram model. **Supplementary Fig. S4:** Estimated scores of 22 immune cell subtypes. **Supplementary Fig. S5:** Differential expression of MHC-related genes.

## Data Availability

The raw data used were obtained from publicly available databases in this study. The specific databases and their accession numbers or URLs are provided in this paper's “Materials and methods” section. The data were accessed on 2023–3 and were available until then. Researchers can access the same data by visiting the respective databases and following the instructions provided by the database administrators. Patients with primary PRAD (n = 3) and paracancerous tissue samples from the same patients (n = 3) were obtained from the Department of Urology, the Fifth Affiliated Hospital of Guangzhou Medical University. Further inquiries can be directed to the corresponding authors.
