# Supplementary material for: Predicting prostate adenocarcinoma patients’ survival and immune signature: a novel risk model based on telomere-related genes
Source: Discov Oncol. 2024 Jun 2;15:203. doi: 10.1007/s12672-024-00986-2 (PMC11144689; doi:10.1007/s12672-024-00986-2)
Supplement: Supplementary file 1 — Additional file 1. Telomere-related prognostic genes Supplementary Table 1: Detailed clinical characteristics of the 500 PRAD patients. Supplementary Fig. S1: PPI graph of TRPGs, PPI: Protein-protein interaction. Supplementary Fig. S2: Enrichment of motifs and transcription factors. Supplementary Fig. S3: DCA analysis curves for nomogram model. Supplementary Fig. S4: Estimated scores of 22 immune cell subtypes. Supplementary Fig. S5: Differential expression of MHC-related genes. [file 12672_2024_986_MOESM1_ESM.pdf]

Supplementary Material

Supplementary Table 1: Detailed clinical characteristics of the 500 PRAD patients.

Supplementary Fig. S1: PPI graph of TRPGs, PPI: Protein-protein interaction.

Supplementary Fig. S2: Enrichment of motifs and transcription factors.

Supplementary Fig. S3: DCA analysis curves for nomogram model.

Supplementary Fig. S4: Estimated scores of 22 immune cell subtypes.

Supplementary Fig. S5: Differential expression of MHC-related genes.

Supplementary File 1: 136 Telomere-related prognostic genes.

TABLE 1 | Basic clinical characteristics of PRAD patients in TCGA-PRAD cohort

Characteristic	Overall (N=500)	High-Risk (N=212)	Low-Risk (N=212)	P-Value (High, Low-Risk)
**Fustat**				0.00703
**Alive**	490 (98.0%)	203 (95.8%)	212 (100%)	
**Death**	10 (2.0%)	9 (4.2%)	0 (0.0%)	
**Age**				0.172
**Mean(SD)**	61.0 (6.82)	61.8 (6.70)	60.9 (6.64)	
**Median (Min, Max)**	61.0 (41.0,78.0)	63.0 (41.0,78.0)	61.0(44.0,77.0)	
**T**				0.0184
**T2**	188 (37.6%)	59 (27.8%)	86 (40.6%)	
**T3**	295 (59.0%)	146 (48.9%)	122 (57.5%)	
**T4**	10 (2.0%)	7 (3.3%)	4 (1.9%)	
**Unknow**	7 (1.4%)			
**N**				<0.001
**N0**	348 (69.6%)	155 (73.1%)	189 (89.2%)	
**N1**	79 (15.8%)	57 (73.1%)	23 (10.8%)	
**Unknow**	73 (14.6%)			

Supplementary Table 1 Detailed clinical characteristics of the 500 PRAD patients.

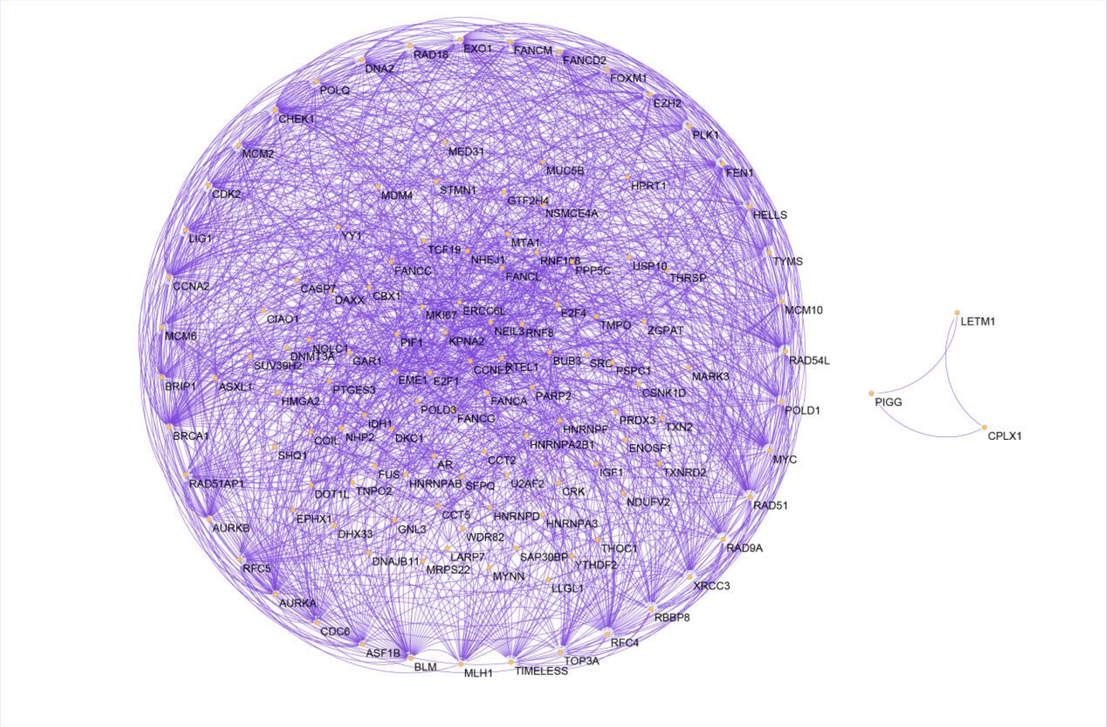

Supplementary Fig. S1 PPI graph of TRPGs, PPI: Protein-protein interaction.

2023/5/16 2.Module_Gene.html

Show 10 entries Search:

	logo	geneSet	motif	NES	AUC	TF_highConf	TF_lowConf	nEnrGenes	rankAtMax	enrichedGenes
	All	All	All	All	All	All	All	All	All	All
1		Module_Gene	cisbp__M0497	7.09	0.434			4	677	BUB3;GTF2H4;LARP7;T
2		Module_Gene	cisbp__M4692	6.48	0.399	SIX5 (directAnnotation).	EGR1; ETS1; HCFC1; SETDB1; SMARCC2; THAP11; ZNF143; ZNF76 (inferredBy_MotifSimilarity).	3	219	BUB3;GTF2H4;TOP3A
3		Module_Gene	cisbp__M6135	6.39	0.394	TBX2 (inferredBy_Orthology).	EGR1; ETS1; SETDB1; SIX5; SMARCC2; STAT5A; STAT5B; THAP11; ZNF143; ZNF76 (inferredBy_MotifSimilarity).	3	219	BUB3;GTF2H4;TOP3A
4		Module_Gene	cisbp__M4484	6.29	0.389	ZNF143 (directAnnotation).	EGR1; ETS1; HCFC1; SETDB1; SIX5; SMARCC2; THAP11; ZNF76 (inferredBy_MotifSimilarity). TBX2 (inferredBy_MotifSimilarity_n_Orthology).	3	219	BUB3;GTF2H4;TOP3A
5		Module_Gene	cisbp__M4527	6.28	0.388	SMARCC2 (directAnnotation).	EGR1; ETS1; HCFC1; SETDB1; SIX5; THAP11; ZNF143; ZNF76 (inferredBy_MotifSimilarity). TBX2 (inferredBy_MotifSimilarity_n_Orthology).	3	219	BUB3;GTF2H4;TOP3A
6		Module_Gene	cisbp__M4618	6.26	0.387	ETS1 (directAnnotation).	EGR1; HCFC1; SETDB1; SIX5; SMARCC2; THAP11; ZNF143; ZNF76 (inferredBy_MotifSimilarity). TBX2 (inferredBy_MotifSimilarity_n_Orthology).	3	219	BUB3;GTF2H4;TOP3A
7		Module_Gene	cisbp__M4461	6.21	0.384	ETS1 (directAnnotation).	EGR1; HCFC1; SETDB1; SIX5; SMARCC2; STAT5A; STAT5B; THAP11; ZNF143; ZNF76 (inferredBy_MotifSimilarity). TBX2 (inferredBy_MotifSimilarity_n_Orthology).	3	219	BUB3;GTF2H4;TOP3A
8		Module_Gene	cisbp__M3134	6.17	0.382	E2F1 (directAnnotation).	E2F2; E2F3; E2F4; E2F5; E2F7; E2F8; RB1; TFDP1 (inferredBy_MotifSimilarity).	3	282	BUB3;HELLS;LARP7
9		Module_Gene	cisbp__M6193	6.17	0.382	E2F4 (directAnnotation).	E2F1; E2F3; E2F7; E2F8; TFDP1 (inferredBy_MotifSimilarity).	3	219	BUB3;HELLS;LARP7
10		Module_Gene	cisbp__M4693	5.9	0.366	SIX5 (directAnnotation).		3	510	BUB3;GTF2H4;TOP3A

Showing 1 to 10 of 97 entries Previous 1 2 3 4 5 … 10 Next

file:///F:/生信/project-多数据集探讨前列腺癌端粒相关分子机制研究-0112/12.Motif prediction/2.Module_Gene.html 1/2

Supplementary Fig. S2 Enrichment of motifs and transcription factors.

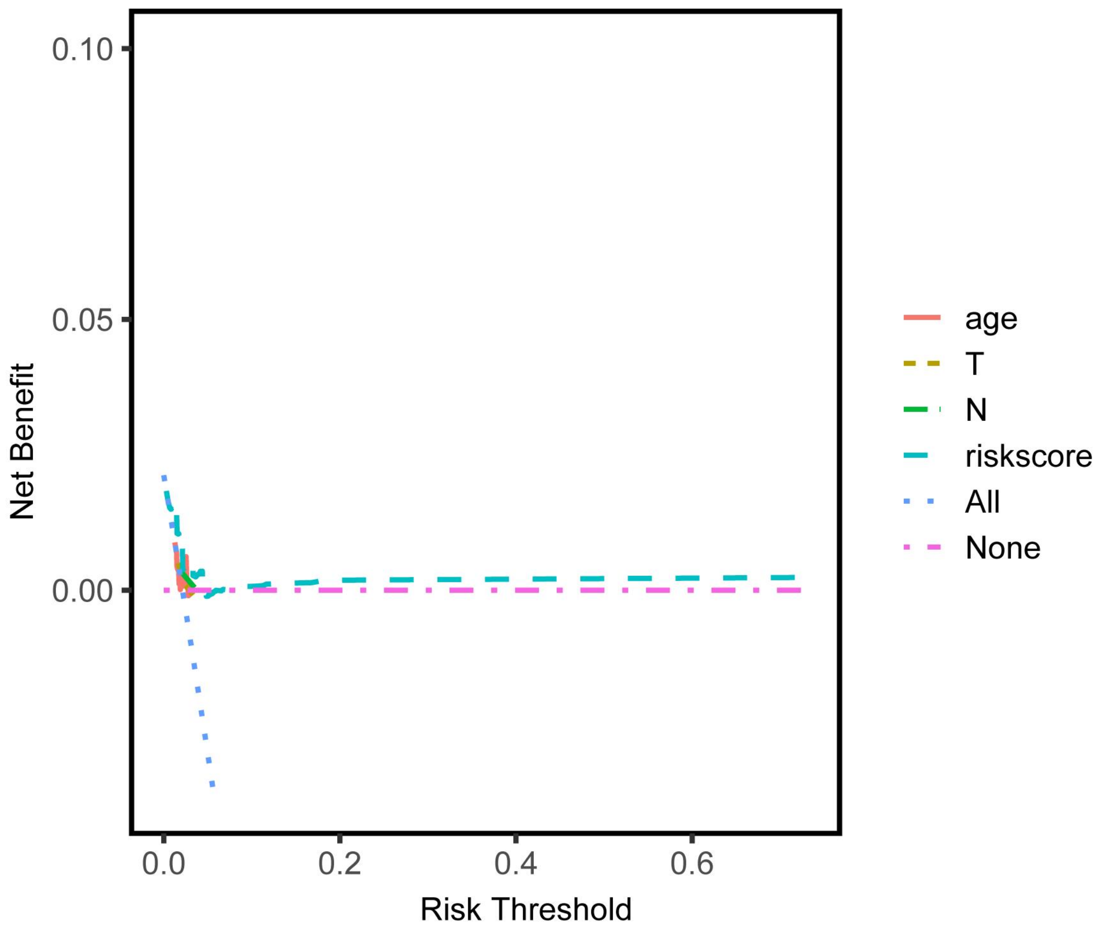

Supplementary Fig. S3　DCA analysis curves for nomogram model.

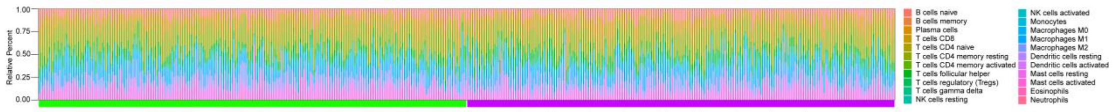

Supplementary Fig. S4. Estimated scores of 22 immune cell subtypes.

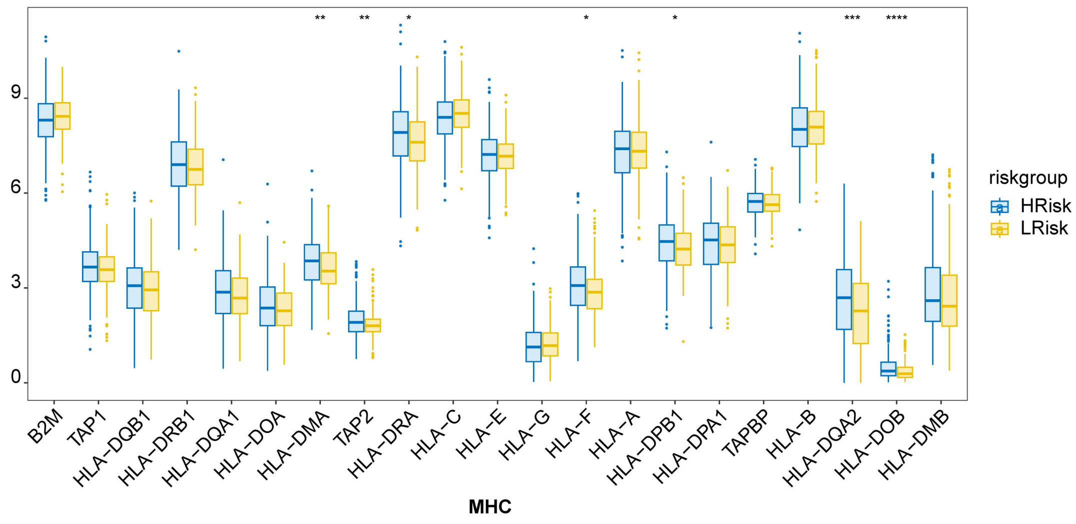

Supplementary Fig. S5   Differential expression of MHC-related genes.

Supplementary file 1: 136 Telomere-related prognostic genes

gene	HR	z	pvalue	lower	upper
HMGA2	2.489872408	4.174536597	2.99E-05	1.622445583	3.821061658
GTF2H4	2.231253697	3.96053237	7.48E-05	1.499896126	3.319225227
TOP3A	2.616939795	3.757530919	0.000171598	1.5844135	4.322340027
POLD1	1.92773076	3.615518633	0.000299747	1.350589657	2.751498848
FANCA	2.047675983	3.489081363	0.000484684	1.369029125	3.062737566
POLQ	1.902039485	3.485226202	0.000491721	1.324941364	2.730501364
NEIL3	1.866739628	3.476165202	0.000508639	1.312921538	2.654169909
HELLS	1.841870456	3.463998497	0.000532209	1.303685748	2.602227403
EME1	1.625386399	3.460753317	0.000538666	1.234478102	2.140079231
RAD54L	1.80424847	3.44862261	0.000563454	1.290134018	2.523235954
BRIP1	1.732592858	3.39934127	0.000675484	1.262033435	2.378604184
CSNK1D	3.342745735	3.296061979	0.000980504	1.630990888	6.851018687
RAD9A	2.497319343	3.253502702	0.001139916	1.438894998	4.334300913
THRSP	1.278566878	3.242198594	0.001186113	1.102065269	1.483336158
SUV39H2	1.77852035	3.218206713	0.001289948	1.252469311	2.525518675
GNL3	3.611165527	3.160207877	0.001576566	1.628523167	8.007571969
SRC	2.58695241	3.159516763	0.00158031	1.434566383	4.66504921
LIG1	1.981867825	3.152765107	0.001617319	1.295369678	3.032184668
CHEK1	1.873539835	3.151294899	0.001625483	1.267881971	2.768515992
CDK2	2.451742637	3.126709842	0.001767743	1.397443652	4.301455697
XRCC3	2.221610759	3.119112676	0.001813966	1.345338042	3.668635102
U2AF2	3.061500745	3.115596723	0.001835731	1.514405194	6.189087868
RBBP8	3.066262713	3.109826789	0.001871971	1.513298118	6.212898115
SAP30BP	2.535344123	3.066603479	0.002165059	1.398955983	4.594833504
RFC5	2.158786807	3.045018511	0.00232666	1.315501947	3.542648103
BUB3	2.191265682	3.040856315	0.002359064	1.321606867	3.633187302
UPF3A	2.610039959	3.034321796	0.002410771	1.404499664	4.850345471
RNF8	2.592590622	3.0250533	0.002485893	1.3985286	4.806141347
MCM10	1.700070715	3.004382334	0.002661207	1.202590076	2.403346322
FUS	3.656366453	3.004071193	0.00266393	1.569263417	8.519293508
BLM	1.701812426	2.974923221	0.002930619	1.198893222	2.415699315
EXO1	1.789851698	2.966827133	0.003008901	1.218426851	2.629266664
DKC1	2.287835719	2.952034274	0.003156879	1.320655321	3.963329564
CCNA2	2.053971295	2.903086218	0.003695049	1.263432861	3.339154942
LARP7	2.110515516	2.89185507	0.003829746	1.272134928	3.501417692
MTA1	2.646791749	2.87684265	0.004016758	1.363719466	5.137058417
HPRT1	2.116748554	2.864740808	0.004173507	1.267233887	3.535751755
MYNN	1.825972105	2.833686482	0.004601445	1.204003058	2.769240581
PTGES3	2.189858279	2.829696646	0.004659216	1.272418327	3.768791425
MUC5B	1.516013586	2.829572876	0.004661018	1.136410513	2.022418101
PFAS	2.199469704	2.812265675	0.004919385	1.269827977	3.809702626
AURKA	1.863074425	2.80462776	0.005037472	1.206103716	2.877900354
RAD18	2.186390093	2.779096154	0.005451039	1.259313735	3.795957676
PIF1	1.59171934	2.765083685	0.005690819	1.144928108	2.21286423
EZH2	2.012598378	2.752132915	0.005920848	1.223018133	3.311931459
RAD51	1.657003162	2.737752941	0.006186053	1.154274834	2.378687811
DOT1L	2.670026831	2.706144952	0.006806933	1.311008825	5.43783012
RTEL1	1.785776442	2.706090828	0.006808042	1.173367592	2.717816244
MED31	2.378697586	2.690591538	0.007132546	1.265303058	4.4718158
EPHX1	0.382446065	-2.684639455	0.007260809	0.189591512	0.771474371

SFPQ	2.283981246	2.683138852	0.00729347	1.249329097	4.175497347
DNA2	2.114464186	2.620752373	0.008773596	1.207799944	3.701737872
RAD51AP1	1.898228416	2.611962167	0.009002422	1.173495328	3.070545772
DNAJB11	2.006370028	2.607211685	0.009128291	1.188705841	3.38647338
HNRNPAB	2.630698048	2.604626517	0.009197446	1.270487483	5.447178592
TYMS	1.860638025	2.571504747	0.010125763	1.15912286	2.986718645
PIGG	2.305456621	2.515284409	0.011893639	1.202510546	4.420027957
ERCC6L	1.646952238	2.507600139	0.012155413	1.115118657	2.432433228
PARP2	1.897052526	2.501080716	0.012381496	1.148585552	3.133252269
RNF168	2.231118847	2.492774932	0.012674916	1.187118231	4.193256559
DHX33	1.985612656	2.485705072	0.012929507	1.156129352	3.410221888
MDM4	1.828136557	2.456233239	0.0140402	1.129633317	2.958555862
CDC6	1.823777096	2.451807779	0.014214057	1.128111748	2.94843388
CIAO1	1.745857545	2.447817261	0.014372453	1.117461786	2.727626665
TXN2	0.481294813	-2.439735813	0.014698006	0.267470515	0.866056943
YY1	2.054334692	2.439621056	0.014702676	1.152058922	3.663259705
TIMELESS	2.022574713	2.421038054	0.015476257	1.1435573	3.577265847
THOC1	1.966410606	2.406058634	0.016125676	1.133570635	3.411142236
TCF19	1.892315375	2.392589864	0.016729931	1.122239914	3.190812796
NSMCE4A	2.725980686	2.374050968	0.017594122	1.191145466	6.238508151
C9orf78	1.832231467	2.371100147	0.017735225	1.110706703	3.022465012
FANCM	2.027022183	2.354704351	0.018537448	1.125748724	3.649854398
FANCC	1.632889482	2.345410898	0.019006121	1.083920696	2.459892197
USP10	2.622437367	2.344991997	0.019027488	1.17151504	5.870328173
DNMT3A	2.033583543	2.336874158	0.019445728	1.121292763	3.688119787
FANCD2	1.791493554	2.33389984	0.019600967	1.097918179	2.923213418
MARK3	1.671234122	2.332137824	0.019693442	1.085408881	2.573245475
POLD3	2.12955844	2.326166772	0.020009656	1.126371956	4.026218094
MLH1	2.16053389	2.320668109	0.020304763	1.127200611	4.141149895
NOLC1	2.125149007	2.319117693	0.020388656	1.123832683	4.018621606
PSPC1	1.797625732	2.312350492	0.020758376	1.093489053	2.955181182
HNRNPD	2.001368165	2.29451367	0.021761011	1.106457494	3.620088937
FEN1	1.950871149	2.291865169	0.021913425	1.101615535	3.454833489
HNRNPF	2.786777978	2.289949996	0.022024215	1.159151152	6.699843662
RFC4	1.801348789	2.284035846	0.022369424	1.087090389	2.984901248
PLK1	1.640129032	2.267828287	0.023339675	1.069474213	2.515276395
E2F4	2.317302691	2.24756541	0.024603915	1.113534494	4.82238475
BRCA1	1.61503091	2.247005333	0.024639686	1.063148287	2.453397022
CCT2	2.20286994	2.24672413	0.024657663	1.106056489	4.387331046
SHQ1	2.275334794	2.240751372	0.025042184	1.108513947	4.670350281
FANCL	1.885478906	2.236706716	0.025305515	1.081626477	3.286745267
ENOSF1	2.148738555	2.226956751	0.025950164	1.096038791	4.212512747
ASXL1	1.996233721	2.222422857	0.026254742	1.085059898	3.672561372
TXNRD2	0.610437261	-2.212167915	0.026955065	0.39420363	0.945282137
HNRNPA2B1	2.350938708	2.207240591	0.027297255	1.100500058	5.022183112
MCM2	1.931967689	2.202239322	0.027648408	1.075136862	3.471650247
PKP4	1.933981231	2.193038937	0.028304574	1.072615295	3.487068866
ASF1B	1.819917377	2.189555179	0.028556513	1.064800773	3.110534235
E2F1	1.737681806	2.180315072	0.029234115	1.057431695	2.855539582
COIL	1.952576636	2.177536042	0.029440596	1.069144928	3.565985695
AURKB	1.625340032	2.17386994	0.029714904	1.048954466	2.518441271
TMPO	1.834831924	2.173331413	0.029755383	1.061398918	3.171859451

Supplementary File S1. 136Telomere-relatedprognosticgenes.
